# Inter- and intra-unit reliability of the COSMED K5: Implications for multicentric and longitudinal testing

**DOI:** 10.1371/journal.pone.0241079

**Published:** 2020-10-23

**Authors:** Kay Winkert, Rupert Kamnig, Johannes Kirsten, Jürgen M. Steinacker, Gunnar Treff

**Affiliations:** Division of Sports and Rehabilitation Medicine, Ulm University, Ulm, Baden-Württemberg, Germany; University of Mississippi, UNITED STATES

## Abstract

**Purpose:**

To evaluate the intra-unit (REL_INTRA_) and inter-unit reliability (REL_INTER_) of two structurally identical units of the metabolic analyser K5 (COSMED, Rome, Italy) that allows to utilize either breath-by-breath (BBB) or dynamic mixing chamber (DMC) technology.

**Methods:**

Identical flow- and gas-signals were transmitted to both K5s that always operated simultaneously either in BBB- or DMC-mode. To assess REL_INTRA_ and REL_INTER_, a metabolic simulator was applied to simulate four graded levels of respiration. REL_INTRA_ and REL_INTER_ were expressed as typical error (TE%) and Intraclass Correlation Coefficient (ICC). To assess also inter-unit differences via natural respiratory signals, 12 male athletes performed one incremental bike step test each in BBB- and DMC-mode. Inter-unit differences within biological testing were expressed as percentages.

**Results:**

In BBB, TE% of REL_INTRA_ ranged 0.30–0.67 vs. REL_INTER_ 0.16–1.39 and ICC ranged 0.57–1.00 vs. 0.09–1.00. In DMC, TE% of REL_INTRA_ ranged 0.38–0.90 vs. REL_INTER_ 0.03–0.86 and ICC ranged 0.22–1.00 vs. 0.52–1.00. Mean inter-unit differences ranged -2.30–2.20% (Cohen’s ds (ds) 0.13–1.52) for BBB- and -0.55–0.61% (ds 0.00–0.65) for DMC-mode, respectively. Inter-unit differences for $\dot{V}O_{2}$ and RER were significant (p < 0.05) at each step.

**Conclusion:**

Two structurally identical K5-units demonstrated accurate REL_INTRA_ with TE < 2.0% and similar REL_INTER_ during metabolic simulation. During biological testing, inter-unit differences for $\dot{V}O_{2}$ and RER in BBB-mode were higher than 2% with partially large ES in BBB. Hence, the K5 should be allocated personally wherever possible. Otherwise, e.g. in multicenter studies, a decrease in total reliability needs to be considered especially when the BBB-mode is applied.

## Introduction

Cardiopulmonary exercise (CPX) testing is frequently used to evaluate fitness in healthy and unhealthy populations in cross-sectional [1] or longitudinal [2] evaluations or studies, as underlined by 3357 hits on the search item “$\dot{V}O_{2}\max$ testing” in pubmed within 5 years (Aug. 2020). As for any scientific method, reliability is an important quality measure also for CPX devices and if testing is conducted within one laboratory and only one CPX-unit is applied, the overall variability of one unit between two or more trials is a key quality measure. This will be called intra-unit reliability (i.e. intra-rater reliability [3]) hereafter. As soon as more than one unit of a particular device is used–be that within one or in multiple labs–the variability between two or more CPX-units needs to be considered, too. This will be called inter-unit reliability (i.e. inter-rater reliability [3]) hereafter. A third measure used throughout this paper is the difference between two devices at a given intensity or stage. This will be called inter-device difference hereafter.

Interestingly, reliability of CPX devices has been determined in several studies, with differences ranging 0.12–8.15% for common respiratory variables [4, 5], but these results are nearly always based on results obtained by a single apparatus. However, they are in fact generalized, assuming that a particular unit represents the whole “population” of the respective device. In the rare cases where inter-unit variability between two units of a particular device has been reported, a considerable variability of 0.4–2.1% was calculated [6, 7]. These studies applied either simulated testing [7] via metabolic simulator [8] or biological testing via exercise tests in humans [6]. Of note, metabolic simulators exclude biological variation, but the price to pay is a questionable transferability to human individuals, where expired gas concentrations, their temperature, and humidity may change throughout the test, as respiratory frequency and tidal volumes do, too. Therefore, an integrative approach has been suggested to evaluate quality measures of CPX devices, where technical and biological testing are combined [9, 10].

Based on this approach, we provided intra-unit reliability and validity data for the portable CPX analyzer COSMED K5 (COSMED, Rome, Italy) [11], which allows to select between breath-by-breath (BBB) or dynamic micro mixing chamber (DMC) technology. We found significant different measures of validity between modes (0.03–11.56%), but did not evaluate inter-unit reliability. Furthermore, since other studies have reported differences in reliability and validity for several devices applying either BBB- or DMC-technology [12–14], we hypothesized that inter-unit reliability might also be mode-dependent in the K5. However, to the best of our knowledge, there are no data available that account for intra- and inter-unit reliability of the two measurement principles of the K5.

We therefore aimed to study intra- and inter-unit reliability of the K5 both in BBB- and DMC-mode at low to high ventilation and gas exchange rates under artificial and natural conditions.

## Materials and methods

We used the integrative approach suggested by [9, 10] to evaluate intra- and inter-unit reliability via metabolic simulations, allowing for repeated measurements without any biological variation or influence (study 1). To substantiate our results, we evaluated the differences between two units in biological tests under natural respiration conditions (study 2).

During all measurements, two K5-units operated simultaneously, receiving the identical flow and gas signals (see: *Equipment*). Experiments for metabolic simulation were conducted within one day. Biological testing consisted of two tests per participant, which were conducted within four days, with at least one day off. All experiments were conducted in a well-ventilated laboratory with temperature and humidity ranging from 18–21° C and 60–75%, respectively.

### Participants

Twelve trained to well-trained [15] experienced male triathletes, cyclists, or rowers, all of them regularly incorporating cycling into their training (age 29±3 years, $\dot{V}O_{2}\mathrm{peak}$ 58.0±6.7 mL∙kg^-1^∙min^-1^, measured with a K5 in DMC-mode) participated in the study. They were experienced with CPX testing and were carefully instructed and familiarized with the specific test procedures. Participants were instructed to keep a standardized diet and to avert from strenuous exercise for 24 hours preceding the tests. All participants gave their informed written consent to participate in the study, which was approved by the ethical review board of Ulm University (#123) and was conducted in accordance with the Declaration of Helsinki.

### Equipment

#### Metabolic analyzer K5

All tests were conducted with two units of the metabolic analyzer K5 (firmware 1.2, COSMED, Rome, Italy). The K5 combines a 2 mL dynamic micro mixing chamber with proportional micro sampling technology and a dual gas sampling system (IntelliMET™), allowing for either BBB or DMC measurements. For technical details see [11].

**Fig 1:**
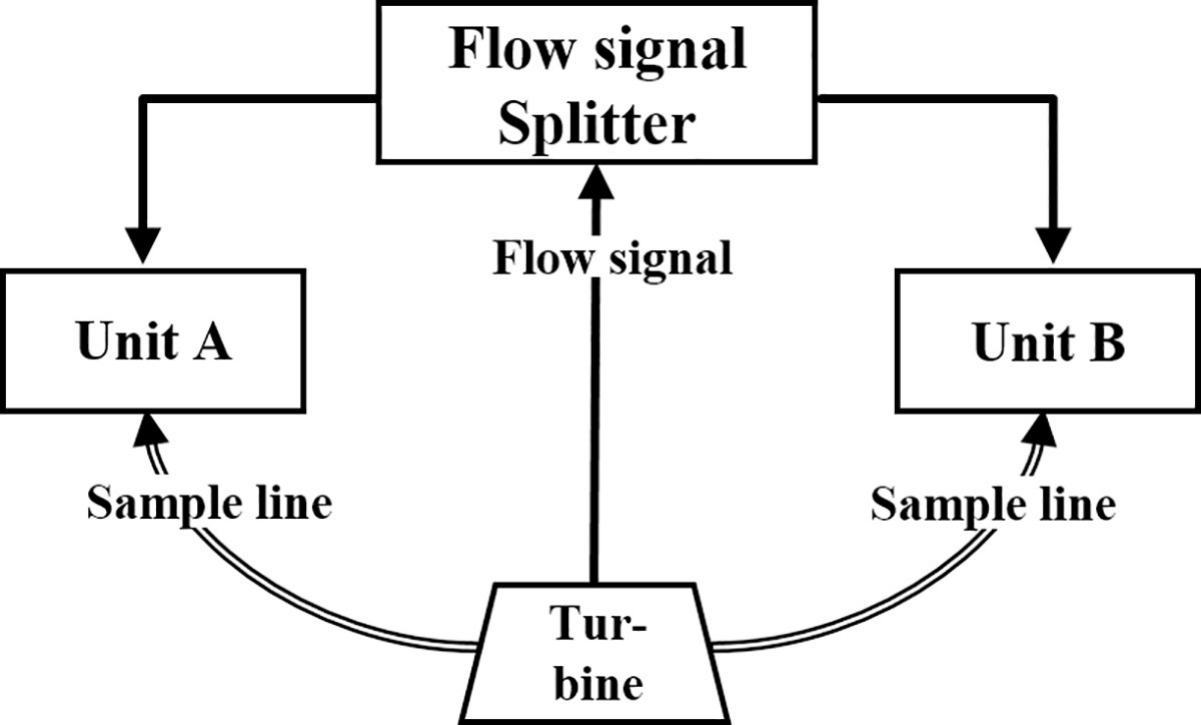
Test setup with simultaneous operation of two identical K5 units using a flow signal splitter.

To allow for simultaneous measurements, both K5s were connected with a single flow sensor via a customized flow signal splitter and both gas sample lines were connected to a customized turbine housing (Fig 1). Except for the flow signal, both devices operated independently and autonomously.

After a 60-min warm-up of the K5s, calibration of flow (simultaneously), gas sensors, and time-delay were conducted for both units according to the manufacturer’s instructions [16].

#### Metabolic simulator

We used a metabolic simulator (MS) (Model: 17056, VacuMed, Ventura, CA, USA) that allows to simulate different measures of gas exchange (oxygen uptake ($\dot{V}O_{2}$), carbon dioxide production ($\dot{V}CO_{2}$); accuracy ±1.00%) and minute ventilation ($\dot{V}E$) by combining different stroke volumes (accuracy ±0.50%), stroke frequencies (accuracy ±1 stroke) and titration of a dry reference gas (21.0% CO_2_, 79.0% nitrogen) with partially tempered, humidified room air, known as the dilution method [8].

#### Cycling ergometer

All exercise tests were conducted on an adjustable electrically braked cycling ergometer (Lode Excalibur, Groningen, Netherland) that was checked for validity prior to the study. Mean differences to calibration device were -1.59%, 95% CI [-2.14, -1.03]) and therefore systematic, without relevance for this study.

### Measuring procedures

#### Study 1 –intra- and inter-unit reliability via metabolic simulation

**Fig 2:**
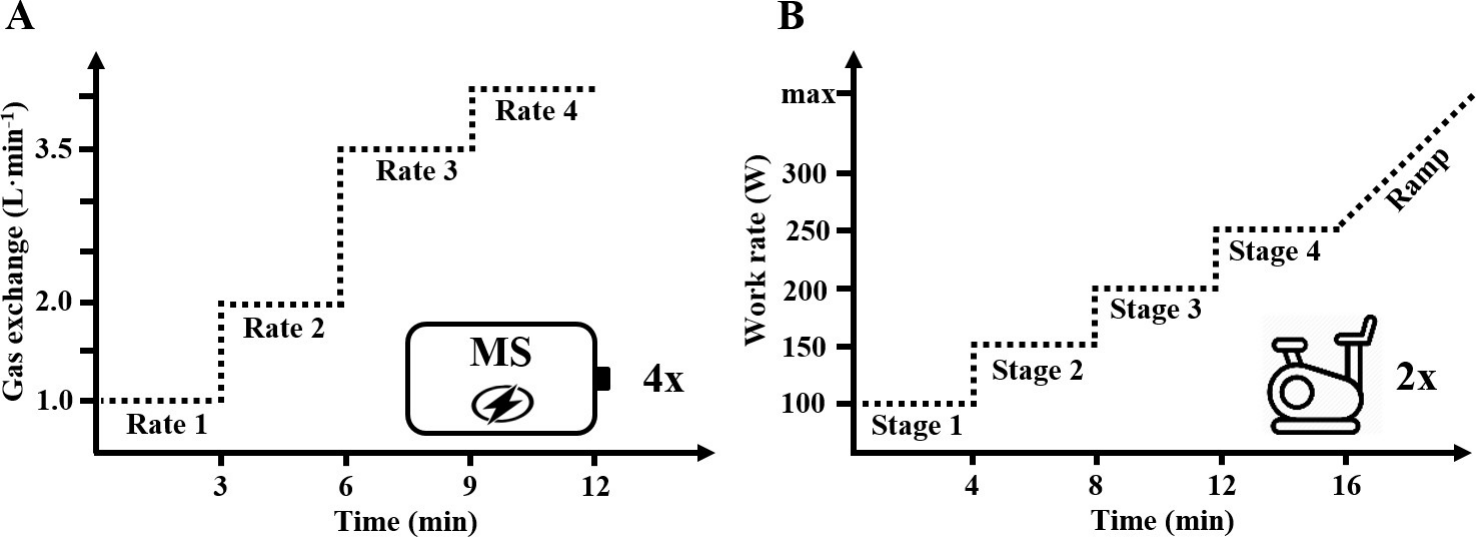
Test protocols for simulated (A, study 1) and biological (B, study 2) testing. To evaluate intra- and inter-unit reliability, study 1 consisted of four metabolic rates produced by a metabolic simulator (MS), conducted four times for breath-by-breath and four times for dynamic mixing chamber mode (left part A). To evaluate inter-unit differences, study 2 consisted of four increasing stages with a consecutive ramp test in 12 male athletes (right part B). The test was conducted twice with both units operating in a randomized order between participants either both in breath-by-breath or both in dynamic mixing chamber mode.

To study intra- and inter-unit reliability via metabolic simulations, a total of eight trials were conducted, each consisting of four increasing steady-state metabolic rates produced by the MS (Bf 20–60 min^-1^, $\dot{V}E$ 30–150 L∙min^-1^, $\dot{V}O_{2}$ 0.94–3.96 L∙min^-1^). Data were recorded with both units operating four times simultaneously in BBB- and four times simultaneously in DMC-mode (Fig 2A).

#### Study 2 –inter-unit differences via biological testing

To study inter-unit differences via biological testing, participants performed two incremental tests within four days, each separated by at least one day. Both tests consisted out of four 4-min stages at 100, 150, 200, 250 W with a consecutive ramp test (starting at 250 W, increment of 40 W∙min^-1^) up to voluntary exhaustion (Fig 2B). Each trial was used to obtain respiratory data from both K5s operating simultaneously either in BBB- or DMC-mode in a randomized order between participants. A standardized warm-up (10 min at 60 W and 5 min at 100 W) preceded each test.

### Data processing and statistics

Respiratory data were calculated by 60-s arithmetic means over the final minute of each rate (study 1) or stage (study 2) based on the exported csv-files. To identify corresponding absolute time points of each unit, markers were manually set during measurements and exported afterwards. Since the MS produced a partially humidified gas mixture at ambient temperature, but the K5 calculates standard temperature and pressure, dry (STPD) conditions, a correction for simulated gas exchange data was applied using a modified spreadsheet [17]. In the consecutive ramp test $\dot{V}O_{2}\mathrm{peak}$ was calculated as the highest 30-s moving average (BBB) or the highest single $\dot{V}O_{2}$-value (DMC), the latter equaling to a 30-s rolling average reported in 10-s intervals.

Overall intra- and inter-unit reliability were calculated by (i) the typical error ($TE\%={CV}_{difference\;score}/\sqrt{2}$) [18], (ii) minimal detectable change $\left(MDC\%=1.96\times TE\%\times \sqrt{2}\right)$ [7], and (iii) intra class correlation coefficient (ICC) [3] using pooled data of all trials and rates of *simulated testing* (*N* = 4 x 4 per unit and mode). According to Koo and Li [10], the intra-unit ICC is defined as a single-measurement, absolute-agreement, 2-way mixed effects model, and the inter-unit ICC by a single-rater, absolute-agreement, 2-way random model. To compare intra-unit A vs. intra-unit B and intra- vs. inter-unit reliability we used the 95% CI of TE%, MDC% and ICC.

To assess detailed differences between devices at particular workloads during exercise testing, we calculated percentage inter-unit differences of biological data at each stage (N = 12 x 5 per unit and mode). Inter-unit differences were also applied to construct Bland-Altman plots displaying magnitude and nature of the inter-unit differences as percentage 100×(*K*5_*A*_−*K*5_*B*_)÷(*K*5_*A*_+*K*5_*B*_/2) with 95% limits of agreement (*differences*±(1.96×*SD*)) [19]. In case of a magnitude dependency, Bland-Altman plots [19] were adapted by means of linear regression analysis and 95% prediction bands. Bland-Altman plots were interpreted according to Atkinson et al. [8], who distinguish between random, systematic, and proportional differences. There are some cut-off values, which are supposed to be used for classifying reliability of metabolic analyzers. Due to the lack of a generally accepted guideline, we applied the most restrictive limits for reliability suggested by Hodges et al. [20], with TE% and inter-unit differences within ±2.00% to be rated as “accurate” for $\dot{V}E$, $\dot{V}CO_{2}$, $\dot{V}O_{2}$, and RER.

Statistical analyses were conducted using SPSS (IBM Corp. Released 2017. IBM SPSS Statistics for Windows, Version 25.0. Armonk, NY: IBM Corp.) unless otherwise stated. Level of significance was set to P ≤ 0.05, distribution and normality of the data were assessed using histograms, probability plots, and Shapiro-Wilks’ tests.

## Results

### Study 1 –intra- and inter-unit reliability via metabolic simulation

Table 1 presents the simulation results for intra- and inter-unit reliability of BBB- and DMC-mode. We found a high and accurate technical intra-unit reliability below 1% in both devices and modes. Differences in reliability of intra-unit reliability between unit A and B were neither significant in BBB- nor in DMC-mode (indicated by 95% CI of TE% and MDC%).

**Table 1:** Study 1. Intra- and inter-unit reliability of two structurally identical units of the metabolic analyzer COSMED K5 in breath-by-breath or dynamic mixing chamber mode during metabolic simulation.

Variable	Unit	Breath-by-breath			Dynamic micro mixing chamber		
		TE% [95% CI]	MDC% [95% CI]	ICC [95% CI]	TE% [95% CI]	MDC% [95% CI]	ICC [95% CI]
$\dot{V}E$	A	0.57 [0.37, 1.16]*	1.57 [1.04, 3.21]*	1.00 [1.00, 1.00]	0.90 [0.59, 1.83]*	2.48 [1.64, 5.08]*	1.00 [1.00, 1.00]
	B	0.49 [0.32, 1.00]^†^	1.36 [0.90, 2.78]^†^	1.00 [1.00, 1.00]	0.90 [0.59, 1.83]^†^	2.48 [1.64, 5.08]^†^	1.00 [1.00, 1.00]
	A vs. B	0.16 [0.11, 0.24]*^†^	0.43 [0.32, 0.67]*^†^	1.00 [1.00, 1.00]	0.03 [0.02,0.05]*^†^	0.08 [0.06,0.13]*^†^	1.00 [1.00, 1.00]
$\dot{V}CO_{2}$	A	0.40 [0.26, 0.81]	1.10 [0.73, 2.25]	1.00 [1.00, 1.00]	0.60 [0.40, 1.22]	1.66 [1.10, 3.39]	1.00 [1.00, 1.00]
	B	0.47 [0.31, 0.96]	1.31 [0.86, 2.67]	1.00 [1.00, 1.00]	0.85 [0.56, 1.74]	2.35 [1.56, 4.82]	1.00 [1.00, 1.00]
	A vs. B	0.82 [0.61, 1.27]	2.28 [1.68, 3.53]	1.00 [0.99, 1.00]	0.86 [0.64, 1.34]	2.40 [1.77, 3.72]	1.00 [1.00, 1.00]
$\dot{V}O_{2}$	A	0.64 [0.42, 1.30]	1.76 [1.17, 3.6]	1.00 [1.00, 1.00]	0.54 [0.36, 1.10]	1.49 [0.98, 3.04]	1.00 [1.00, 1.00]
	B	0.56 [0.37, 1.14]	1.55 [1.03, 3.17]	1.00 [1.00, 1.00]	0.81 [0.54, 1.67]	2.26 [1.49, 4.62]	1.00 [1.00, 1.00]
	A vs. B	1.48 [1.09, 2.29]	4.09 [3.02, 6.36]	1.00 [1.00, 1.00]	0.86 [0.63, 1.33]	2.37 [1.75, 3.68]	1.00 [1.00, 1.00]
RER	A	0.67 [0.44, 1.38]	1.86 [1.23, 3.81]	0.57 [0.11, 0.95]	0.38 [0.25, 0.78]	1.06 [0.70, 2.16]	0.22 [0.00, 0.83]
	B	0.30 [0.20, 0.62]^†^	0.84 [0.56, 1.72]^†^	0.90 [0.54, 0.99]	0.54 [0.36, 1.10]	1.50 [0.99, 3.06]	0.45 [0.05, 0.93]
	A vs. B	1.39 [1.02, 2.16]^†^^#^	3.85 [2.84, 5.99]^†^ ^#^	0.09 [-0.14, 0.42]	0.60 [0.45, 0.94] ^#^	1.67 [1.23, 2.59] ^#^	0.52 [0.07, 0.80]

Intra- and inter-unit reliability based on data of two COSMED K5 units operating simultaneously either in breath-by-breath or dynamic mixing chamber mode during four trials of four incremental simulated metabolic rates using a metabolic simulator (N = 4 x 4 per unit and mode). Intra-unit reliability is given for each unit (A, B). Inter-unit reliability calculated for both units (A vs. B).

TE% = typical error as CV, MDC% = minimal detectable change

* indicates significantly (p ≤ 0.05) different intra-unit A vs. inter-unit A vs. B reliability

^†^ indicates significantly (p ≤ 0.05) different intra-unit B vs. inter-unit A vs. B reliability

^#^ indicates significant (p ≤ 0.05) difference of breath-by-breath vs. dynamic mixing chamber mode

As expected, inter-unit reliability was lower than intra-unit reliability, but always remained within the 2% threshold. While TE%s for inter- vs. intra-unit reliability were only slightly higher in DMC-mode, differences for inter- vs. intra-unit reliability were higher in BBB-mode and significant for RER compared to unit B. Further, intra- vs. inter-unit reliability for $\dot{V}E$ differed significantly between both units and also between BBB- and DMC-mode.

### Study 2 –inter-unit differences via biological testing

**Fig 3:**
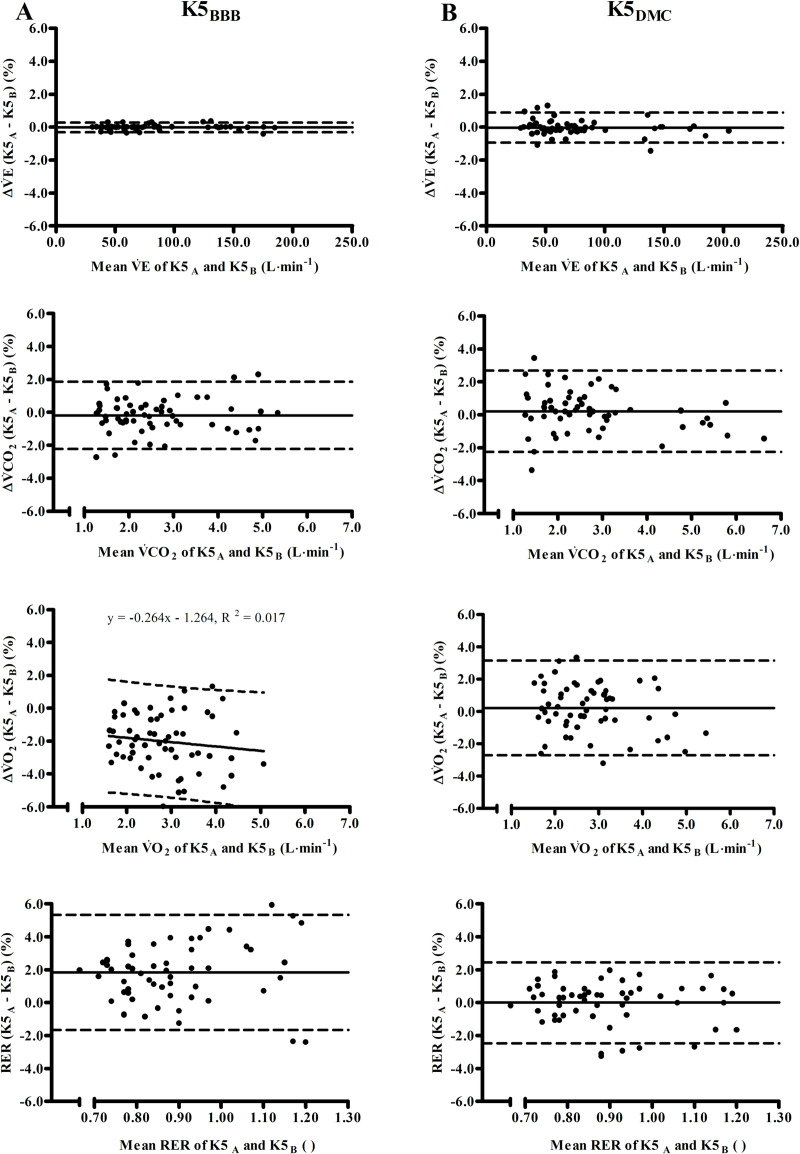
Bland-Altman plots showing differences between two identical K5-units (COSMED, Rome, Italy). Both units operated simultaneously either in breath-by-breath (BBB, left part A) or dynamic mixing chamber (DMC, right part B) mode. Y-axis is 100×(*K*5_*A*_−*K*5_*B*_)÷(*K*5_*A*_+*K*5_*B*_/2). The corresponding mean of both units is shown on the X-axis. Solid and broken lines indicate mean difference and 95% limits of agreement or, in case of magnitude dependent differences, linear regression analysis and 95% prediction intervals, respectively. $\dot{V}E$ = minute ventilation (BTPS), $\dot{V}CO_{2}$ = carbon dioxide production (STPD), $\dot{V}O_{2}$ = oxygen uptake (STPD), RER = respiratory exchange ratio (STPD).

The Bland-Altman-plots in Fig 3 visualize the inter-unit differences between both units in BBB- and DMC-mode. In BBB-mode differences between both units for $\dot{V}E$ and $\dot{V}CO_{2}$ were random, for RER differences were random systematic, while they were rated as proportional systematic for $\dot{V}O_{2}$. In DMC-mode, differences for all variables were random. Table 2 shows percentage differences between both units at each stage, for BBB ranging -2.30% (95% CI [-3.68, -0.92]) to 2.20% (95% CI [0.58, 3.82]) and for DMC-mode -0.55% (95% CI [-1.15, 0.05]) to 0.61% (95% CI [0.09, 1.13]). Inter-unit differences were significantly for $\dot{V}O_{2}$ (stage 1–4) and RER (stage 3) in BBB-mode, and also significant larger in BBB- compared to DMC-mode.

**Table 2:** Study 2. Inter-unit differences of two simultaneously operating COSMED K5s in breath-by-breath and dynamic mixing chamber mode during incremental exercise testing on a cycle ergometer (N = 12).

Variable	Stage	BBB	Difference K5_A_ –K5_B_				DMC	Difference K5_A_ –K5_B_		
		Mean ±SD	Mean% [95% CI]	*p*	ES		Mean ±SD	Mean% [95% CI]	*p*	ES
$\dot{V}E$ (L∙min^-1^)	1	39.47 ±4.48	-0.02 [-0.11, 0.07]	0.658	0.13		38.47 ±5.77	0.09 [-0.14, 0.31]	0.420	0.24
	2	51.31 ±5.40	-0.02 [-0.11, 0.07]	0.642	0.14		51.05 ±6.27	-0.13 [-0.49, 0.23]	0.455	0.22
	3	65.05 ±6.94	-0.03 [-0.11, 0.06]	0.511	0.20		64.27 ±7.76	0.12 [-0.20, 0.45]	0.424	0.24
	4	85.76 ±14.48	0.02 [-0.09, 0.14]	0.682	0.12		81.22 ±9.87	-0.06 [-0.20, 0.07]	0.322	0.30
	5	151.14 ±19.04	-0.02 [-0.13, 0.09]	0.675	0.12		158.19 ±24.22	-0.24 [-0.65, 0.18]	0.914	0.41
$\dot{V}CO_{2}$ (L∙min^-1^)	1	1.40 ±0.10	-0.10 [-0.85, 0.64]	0.767	0.09		1.45 ±0.16	0.13 [-1.07, 1.33]	0.813	0.07
	2	1.85 ±0.11	-0.22 [-0.81, 0.36]	0.415	0.24		1.95 ±0.17	0.28 [-0.46, 1.02]	0.420	0.24
	3	2.33 ±0.17	-0.34 [-0.99, 0.32]	0.285	0.32		2.47 ±0.21	0.61 [0.09, 1.13]	0.025	0.75
	4	2.95 ±0.32	-0.15 [-0.70, 0.41]	0.577	0.17		3.09 ±0.26	0.45 [-0.26, 1.17]	0.193	0.40
	5	4.53 ±0.51	-0.09 [-0.92, 0.74]	0.813	0.07		5.29 ±0.67	-0.55 [-1.15, 0.05]	0.479	0.65
$\dot{V}O_{2}$ (L∙min^-1^)	1	1.79 ±0.16	-1.61 [-2.36, -0.85]	0.001	1.35		1.75 ±0.13	0.16 [-0.79, 1.11]	0.717	0.11
	2	2.30 ±0.20	-1.84 [-2.62, -1.07]	0.000	1.52		2.23 ±0.16	0.28 [-0.68, 1.24]	0.534	0.19
	3	2.75 ±0.18	-2.16 [-3.35, -0.96]	0.002	1.15		2.70 ±0.20	0.55 [-0.37, 1.46]	0.213	0.38
	4	3.22 ±0.19	-2.14 [-3.34, -0.94]	0.002	1.14		3.16 ±0.11	0.42 [-0.46, 1.31]	0.312	0.31
	5	4.07 ±0.48	-2.30 [-3.68, -0.92]	0.004	1.06		4.45 ±0.50	-0.49 [-1.73, 0.76]	0.422	0.28
RER ()	1	0.79 ±0.07	1.51 [0.73, 2.29]	0.001	1.23		0.83 ±0.07	0.01 [-0.84, 0.86]	0.987	0.00
	2	0.81 ±0.07	1.63 [0.75, 2.52]	0.002	1.17		0.88 ±0.06	0.02 [-0.73, 0.77]	0.956	0.02
	3	0.85 ±0.08	1.85 [0.80, 2.90]	0.003	1.12		0.92 ±0.06	0.01 [-0.76, 0.78]	0.978	0.01
	4	0.92 ±0.11	2.01 [0.73, 3.29]	0.005	1.00		0.98 ±0.07	-0.05 [-0.86, 0.76]	0.899	0.04
	5	1.12 ±0.06	2.20 [0.58, 3.82]	0.012		0.86	1.19 ±0.04	0.05 [-1.03, 1.14]	0.565	0.04

Notes: The levels of significance for differences (100×(*K*5_*A*_−*K*5_*B*_)÷(*K*5_*A*_+*K*5_*B*_/2)) between two K5s operating either in breath-by-breath (BBB) or dynamic mixing chamber mode (DMC) during four incremental stages and a consecutive ramp test of bike exercise are based on one-sample t-test (p > 0.05 non-significant (NS), p ≤ 0.05 *, p ≤ 0.01 **, p ≤ 0.001 ***). Cohan´s d effect size was defined as trivial: 0.0–0.2, small: 0.2–0.6, moderate: 0.6–1.2, large: 1.2–2.0, and very large: 2.0–4.0. $\dot{V}E$ is given at body temperature and pressure, saturated (BTPS). While $\dot{V}CO_{2}$ and $\dot{V}O_{2}$ are given at standard temperature and pressure, dry (STPD). Mean or maximal mechanical power output during stages or ramp were 100 W, 150 W, 200 W, 250 W, or 415 ±59 W, respectively.

## Discussion

The aims of this study were the quantification of intra- and inter-unit reliability of two structural identical COSMED K5 metabolic analyzers in BBB- and DMC-mode at low to maximal respiratory rates and the differences between the analyzers. To evaluate intra- and inter-unit reliability in study 1, two identical units operated simultaneously during four trials of four simulated metabolic rates. In study 2, inter-unit differences were determined, during two trials of bike exercise consisting out of four increasing work rates with a consecutive ramp test. The major findings were a high and accurate technical intra-unit reliability below 1% in both devices and also a high and accurate inter-unit reliability in BBB-mode, albeit almost 75% lower. During biological testing, we found several significant mean differences in $\dot{V}O_{2}$ and RER > 2% between both units operating in BBB (*trivial* to *large*; highest 95% CI range ±3.7%), but < 1% in DMC (*trivial* to *moderate*; highest 95% CI range ±1.8%). Hence, inter-unit differences were occasionally significantly larger in BBB- than in DMC-mode.

### Intra- and inter-unit reliability via metabolic simulation (study 1)

Generally speaking, small variations between two or more units are likely, because calibration, data-acquisition, data processing, and manufacturing tolerances of the hardware add inherent measurement noise. Nevertheless, inter-unit reliability should be close to intra-unit reliability, to prevent that the inaccuracy of an additional device exaggerates the variability of cross-sectional (multicenter studies) and longitudinal (individual monitoring) data in addition to the unavoidable intra-unit variability.

We found an accurate intra-unit reliability of the K5 in both modes and for each respiratory variable, indicated by 95% CI of the TE%s within 2.00% [20] and moderate to excellent ICCs [3]. While the ICCs for intra-unit reliability were almost perfect for $\dot{V}E$, $\dot{V}CO_{2}$, and $\dot{V}O_{2}$ in both modes, ICCs for RER were remarkably low, especially in DMC-mode. This is attributable to the dilution method [8], where the MS produces constant ratios for $\dot{V}CO_{2}$ and $\dot{V}O_{2}$, resulting in a nearly constant RER of approx. 1.00 at all rates and trials. Consequently, variability within and between rates is very low and therefore even slightest alterations will have a distinctive effect on the calculated ICC, even though TE% and MDC% indicate an accurate reliability. Aside from that, an unknown percentage of variability between repeated trials is related to the recalibration of the K5s between the repeated trials and the inaccuracy of the MS. However, intra-unit mean TE%s are below or within the inaccuracy range of the MS, which has been reported as ±0.50–1.00% [17].

Unsurprisingly, except for $\dot{V}E$, which was measured by a single flow sensor, inter-unit reliability of the K5 in both modes tended to be lower than intra-unit reliability, but not significantly. It is worth to mention that inter-unit reliability of RER measured in BBB-mode was significantly lower than intra-unit variability of unit B. Hence, if BBB-mode is applied, the addition of device A to a virtual test setup would definitely increase the variability of longitudinal or cross-sectional RER data in comparison to device B, only. Notably, intra-unit reliability in our study was in line with previous data [11, 13] as well as with very limited data on inter-unit reliability that solely focused on BBB-mode during metabolic simulation [7].

### Inter-unit differences via biological testing (study 2)

The detailed differences for each biological stage between both units in each mode (Table 2 and Fig 2B) indicate that inter-unit differences were always below the 2.00%-threshold [20] for biological testing in DMC-mode including the upper 95% CI. In BBB-mode, the situation was less clear-cut, because while for $\dot{V}E$ and $\dot{V}CO_{2}$ inter-unit differences were always lower than 2.00% [20], differences for $\dot{V}O_{2}$ and RER increased with intensity and upper limits of 95% CI exceeded the 2.00%-threshold up to 1.9-fold (Table 2 and Fig 2A). It is worth to mention that even though mean differences exceeded the 2.00% cut off [20] only slightly, they were significant and of *moderate* to *large* effect size. Hence, biological testing indicated that inter-unit differences could be substantial at certain workloads, even if technical inter-unit reliability is accurate.

To the best of our knowledge the only study that focused inter-unit variability so far, reported mean differences between two identical DMC systems (TrueOne 2400, ParvoMedics, Salt Lake City, USA) of 0.8 to 2.6% at low to moderate intensities on a bike ergometer (30 to 120 W) [6]. Considering the higher exercise intensities of up to 409 W during biological testing in our study, the K5-results for inter-unit differences fit well and indicate superior inter-unit reliability for the DMC-mode.

### Practical implications of intra- and inter-unit reliability

Our results indicate that intra-unit reliability of the COSMED K5 is highly accurate for both modes and the magnitude of inter-unit TE%s are in the similar range. Inter-unit differences for all variables obtained in DMC-mode (-0.55–0.61%) are probably neither of any physiological relevance–assuming a biological variability of ~2.00% [6, 21–24]-, nor of practical relevance—assuming a smallest worthwhile change of about 0.3–1.2% [25–27]. But when using the K5 in BBB-mode, scientists and practitioners should be aware of inter-unit differences for $\dot{V}O_{2}$ and RER of up to -2.30% (95% CI [-3.68, -0.92]) at high intensities, which exceed intra-device reliability and thereby add additional noise. Hence, if possible, researchers should assign a particular unit personalized in longitudinal settings. If that is not possible, e.g., when CPX-data are collected in multicenter studies, we recommend to use the DMC-mode of the K5, due to its lower variability in comparison to the BBB-mode. However, irrespective of the mode, inter-unit reliability and -differences have to be considered because of their impact on the MDC%. The MDC% is practically very relevant, because it represents the smallest detectable change within repeated measurements or the smallest detectable between devices, respectively. In other words, the similar intra- and inter-unit MDC%s in our dataset (e.g. 2.3 vs. 2.4% for $\dot{V}O_{2}$ in DMC) indicate that the technical variability obtained by two measurements with different but structurally identical units is already as high as the variability obtained by four repeated simulated measurements with only one instrument.

Our study has a few potential limitations that need to be mentioned: The relatively short observation period did not allow to evaluate potential impact of the age of the metabolic analyzer on reliability. Furthermore, it should be noted that the results of our biological tests might be limited to cycling ergometer exercise, because we cannot exclude that other types of exercise might influence the results. Finally, the results are strictly speaking limited to male athletes and it should be noted that female athletes were not acquired for this study, because we aimed to stress the K5 with high minute ventilations. These are generally higher in males and therefore potential problems especially in BBB-mode are probably rather mitigated in female athletes.

## Conclusion

Two simultaneously operating COSMED K5s demonstrated accurate intra-unit reliability during a wide range of low to maximal simulated intensities, indicated by TE% < 2.00%, MDC% < 2.48 and ICCs > 0.93 (except for RER). Inter-unit reliability was not significantly lower and we found a considerable mode dependency due to *moderate* to *large* inter-unit differences of 3.7–3.8% for $\dot{V}O_{2}$ and RER measurements in BBB-mode (95% CI), while differences were *small* to *moderate* and not of physiological relevance in DMC-mode. Therefore, inter-unit reliability adds additional variability especially in BBB-mode. We conclude that whenever possible, units of the same K5 model should be assigned personalized in longitudinal studies and if several units are used in multicenter studies, inter-unit reliability needs to be considered because it increases the smallest worthwhile change in cardiopulmonary exercise tests.

## Supporting information

S1 File Raw data. (XLS)
